# Assessment of Out-of-Pocket Spending for COVID-19 Hospitalizations in the US in 2020

**DOI:** 10.1001/jamanetworkopen.2021.29894

**Published:** 2021-10-18

**Authors:** Kao-Ping Chua, Rena M. Conti, Nora V. Becker

**Affiliations:** 1 Department of Pediatrics, Susan B. Meister Child Health Evaluation and Research Center, University of Michigan Medical School, Ann Arbor; 2 Department of Health Management and Policy, University of Michigan School of Public Health, Ann Arbor; 3 Department of Markets, Public Policy, and Law, Institute for Health System Innovation and Policy, Questrom School of Business, Boston University, Boston, Massachusetts; 4 Division of General Medicine, Department of Internal Medicine, University of Michigan Medical School, Ann Arbor

## Abstract

**Importance:**

Many insurers waived cost sharing for COVID-19 hospitalizations during 2020. Nonetheless, patients may have been billed if their plans did not implement waivers or if waivers did not capture all hospitalization-related care. Assessment of out-of-pocket spending for COVID-19 hospitalizations in 2020 may show the financial burden that patients may experience if insurers allow waivers to expire, as many chose to do during 2021.

**Objective:**

To estimate out-of-pocket spending for COVID-19 hospitalizations in the US in 2020.

**Design, Setting, and Participants:**

This cross-sectional study used data from the IQVIA PharMetrics Plus for Academics Database, a national claims database representing 7.7 million privately insured patients and 1.0 million Medicare Advantage patients, regarding COVID-19 hospitalizations for privately insured and Medicare Advantage patients from March to September 2020.

**Main Outcomes and Measures:**

Mean total out-of-pocket spending, defined as the sum of out-of-pocket spending for facility services billed by hospitals (eg, accommodation charges) and professional and ancillary services billed by clinicians and ancillary providers (eg, clinician inpatient evaluation and management, ambulance transport).

**Results:**

Analyses included 4075 hospitalizations; 2091 (51.3%) were for male patients, and the mean (SD) age of patients was 66.8 (14.8) years. Of these hospitalizations, 1377 (33.8%) were for privately insured patients. Out-of-pocket spending for facility services, professional and ancillary services, or both was reported for 981 of 1377 hospitalizations for privately insured patients (71.2%) and 1324 of 2968 hospitalizations for Medicare Advantage patients (49.1%). Among these hospitalizations, mean (SD) total out-of-pocket spending was $788 ($1411) for privately insured patients and $277 ($363) for Medicare Advantage patients. In contrast, out-of-pocket spending for facility services was reported for 63 hospitalizations for privately insured patients (4.6%) and 36 hospitalizations for Medicare Advantage patients (1.3%). Among these hospitalizations, mean (SD) total out-of-pocket spending was $3840 ($3186) for privately insured patients and $1536 ($1402) for Medicare Advantage patients. Total out-of-pocket spending exceeded $4000 for 2.5% of privately insured hospitalizations compared with 0.2% of Medicare Advantage hospitalizations.

**Conclusions and Relevance:**

In this cross-sectional study, few patients hospitalized for COVID-19 in 2020 were billed for facility services provided by hospitals, suggesting that most were covered by insurers with cost-sharing waivers. However, many patients were billed for professional and ancillary services, suggesting that insurer cost-sharing waivers may not have covered all hospitalization-related care. High cost sharing for patients who were billed by facility services suggests that out-of-pocket spending may be substantial for patients whose insurers have allowed waivers to expire.

## Key Points

**Question:**

How much were patients billed for COVID-19 hospitalizations in the US in 2020?

**Findings:**

In this cross-sectional study of 4075 COVID-19 hospitalizations in 2020, 71.2% of privately insured patients and 49.1% of Medicare Advantage patients had cost sharing for any hospitalization-related service, including those billed by clinicians; 4.6% of privately insured and 1.3% of Medicare Advantage had cost sharing for facility services billed by hospitals, with mean out-of-pocket spending of $3840 and $1536, respectively.

**Meaning:**

The findings suggest that out-of-pocket spending for COVID-19 hospitalizations may be substantial if insurers allow cost-sharing waivers to expire.

## Teaser

This cross-sectional study assesses out-of-pocket spending by privately insured and Medicare Advantage patients for COVD-19 hospitalizations in 2020.

## Introduction

From August 2020 through July 2021, there were 2.4 million US hospitalizations for COVID-19.^1^ To mitigate patient financial burden, many private insurers and Medicaid Advantage insurers voluntarily waived cost sharing for COVID-19 hospitalizations during part or all of 2020.^2,3^ The literature examining cost sharing for other respiratory infection–related hospitalizations suggests that these waivers potentially resulted in substantial savings for patients.^4,5,6^ For example, among privately insured patients hospitalized for treatment of respiratory infections between 2016 and 2019, average out-of-pocket spending was $1653 for those in traditional plans and $1961 for those in consumer-driven health plans.^4^ Among Medicare Advantage patients hospitalized for treatment of influenza in 2018, mean out-of-pocket spending was almost $1000.^6^

Although waivers may have mitigated the financial burden for many patients hospitalized for treatment of COVID-19 during 2020, some patients may still have been billed if their plans did not implement waivers or if waivers did not capture all hospitalization-related care. Hospitalizations can result in 2 categories of bills.^7,8^ The first includes facility services provided by hospitals, such as accommodation and inpatient pharmacy services. The second includes services from clinicians and ancillary service providers (hereafter referred to as professional and ancillary services). This category includes clinician services for emergency department and inpatient care as well as ambulance services for transport to the hospital. Although waivers would ideally cover both categories, some may have covered only facility services billed by hospitals, not professional and ancillary services billed separately by professionals providing those services.

Although protecting patients from the costs of hospitalization is an important goal regardless of condition, protecting patients from the costs of COVID-19 hospitalizations specifically may be especially important given the number of hospitalizations that may occur and given that the threat of cost sharing could deter patients with serious COVID-19 symptoms from seeking care. Despite this, to our knowledge, no study has assessed the amount for which patients were billed for COVID-19 hospitalizations during 2020 either overall or by service category. Addressing this knowledge gap may inform policy in several ways. First, it may demonstrate the potential financial burden patients may experience if insurers allow cost-sharing waivers to expire, as many chose to do during 2021.^9,10^ Second, it may motivate efforts to improve the comprehensiveness and implementation of the remaining insurer cost-sharing waivers for COVID-19 hospitalizations. Third, it may indicate the potential need for federal legislation mandating US insurers to waive cost sharing for these hospitalizations; this legislation was proposed but not passed in the US House of Representatives in 2020.^11^ Fourth, it may inform cost-sharing policies for hospitalizations during future pandemics. In this study, we used national claims data to estimate out-of-pocket spending for COVID-19 hospitalizations from March to September 2020 among patients covered by private insurance and Medicare Advantage plans.

## Methods

### Data Source

In May 2021, we conducted a cross-sectional analysis of the IQVIA PharMetrics Plus for Academics database (IQVIA Inc). This database contains fully adjudicated medical and pharmacy claims from deidentified patients in all 50 states and the District of Columbia. Claims were complete through September 30, 2020, at the time of analysis. The database included 1.0 million patients covered by Medicare Advantage plans and 7.7 million patients covered by private plans in 2020, all of which were fully insured plans. Data contributors are a fixed group of plans, the identities of which are confidential. Because data were deidentified, the institutional review board of the University of Michigan Medical School exempted analyses from human participant review. This study followed the Strengthening the Reporting of Observational Studies in Epidemiology (STROBE) reporting guideline for cross-sectional studies.^12^

The database includes patient year of birth, state, payer type, and plan type. The database also includes *International Classification of Diseases, Tenth Revision, Clinical Modification* (*ICD-10-CM*) diagnosis codes, a hospitalization identifier assigned to all claims that occurred on or between the admission and discharge dates of hospitalizations, amounts billed to patients (deductibles, co-insurance, and co-payments), and information regarding whether the billing provider was a hospital, clinician, or other entity. The database does not report race, ethnicity, out-of-network status, or in-hospital death (to protect confidentiality). Moreover, the database does not include plan identifiers or information on plan benefit design, including whether insurers had cost-sharing waivers for COVID-19 hospitalizations or which services such waivers covered. We conducted analyses to evaluate whether waivers may have been in place.

### Study Sample

We included hospitalizations that had a confirmed primary diagnosis of COVID-19 infection (*ICD-10-CM* diagnosis code U071) that began and ended between March 1 and September 29, 2020. We required discharge before September 30, 2020, to ensure that the end of hospitalization was observed (eAppendix 1 in the Supplement gives the details). We excluded hospitalizations if they were covered by a secondary insurer (eg, a different private insurance plan) or if any associated claim had missing data for out-of-pocket spending or billing provider type.

### Categorization of Claims

For each hospitalization, we assigned claims with the corresponding hospitalization identifier to 1 of 3 mutually exclusive categories: facility services, professional and ancillary services, and unclassified services (eAppendix 2 in the Supplement gives details). Claims for facility services were defined as institutional claims with a hospital or emergency department place of service and a hospital billing provider type. These services included but were not limited to hospital accommodation and inpatient laboratory and pharmacy services. Claims for professional and ancillary services were defined as 1 of 3 types of services: ambulance (claims with an ambulance place of service or procedure code), clinician (claims with an emergency department or hospital place of service and clinician billing provider type), and miscellaneous (claims with billing provider type for miscellaneous providers, such as durable medical equipment providers). For additional context, clinician services were divided into 4 subtypes: emergency department (claims with an emergency department place of service), inpatient evaluation and management (claims with a hospital place of service and procedure code for evaluation and management, such as initial hospital care), inpatient diagnostic testing (claims with a hospital place of service and procedure codes for laboratory tests, radiology tests, electrocardiography, echocardiography, electroencephalography, and vascular diagnostic studies), and other inpatient services (claims with hospital place of service and procedure codes for services other than evaluation and management and diagnostic testing, such as procedures). Claims for unclassified services were the 4.3% of claims that were assigned the confinement identifier for the COVID-19 hospitalization but did not meet criteria for a facility or professional or ancillary service. For three-quarters of these claims, the place of service was office, home, or hospital outpatient department. Although some could represent care provided at visits resulting in direct hospital admission, others could represent care provided at unrelated visits. In the main analysis, we excluded these claims to maximize the probability of capturing only out-of-pocket spending for services truly associated with hospitalizations. These claims were included in a sensitivity analysis (eAppendix 3 in the Supplement).

### Outcomes

Out-of-pocket spending was the sum of deductibles, co-insurance, and co-payments; this quantity excluded any surprise bills for out-of-network care.^8^ For each payer type (private insurance and Medicare Advantage), we determined the proportion of hospitalizations in 2 categories: those that had out-of-pocket spending for facility services (with or without out-of-pocket spending for professional and ancillary services) and those that had out-of-pocket spending for facility services, professional and ancillary services, or both. For hospitalizations in both categories, we calculated total out-of-pocket spending, defined as the sum of out-of-pocket spending across facility and professional and ancillary services. In addition, we calculated the proportion of all hospitalizations with out-of-pocket spending for the 3 main types of professional and ancillary services and for the 4 subtypes of clinician services.

### Presence of Cost-Sharing Waivers

The database did not report whether COVID-19 hospitalizations were covered by plans with cost-sharing waivers. However, as noted in the Results section, few hospitalizations in our sample had cost sharing for facility services. Although this might suggest that most hospitalizations were covered by insurers that waived cost sharing for facility services (ie, that the absence of cost sharing for facility services implied the presence of a waiver), a potential alternative explanation is that most patients had already met their plan’s annual out-of-pocket maximum at the time of the hospitalization. To evaluate this possibility, we restricted analyses to hospitalizations of patients continuously enrolled since January 2020, calculated out-of-pocket spending across medical and pharmacy claims in 2020 before the hospitalization, and calculated the incidence of out-of-pocket spending for facility services among hospitalizations for patients in the lowest quartile of this prior out-of-pocket spending. These patients likely had not met out-of-pocket maximums at the time of their hospitalization. If few of these patients had cost sharing for facility services, cost-sharing waivers, rather than meeting out-of-pocket maximums, may have been associated with the low observed incidence of cost sharing for facility services.

We also explored whether it was reasonable to assume that hospitalizations with out-of-pocket spending for facility services were not covered by insurers with cost-sharing waivers for these services (ie, that the presence of cost sharing for facility services implied the absence of a waiver—the inverse of the previously mentioned assumption). To evaluate this assumption, we compared the incidence of out-of-pocket spending for facility services between hospitalizations for COVID-19 and those for influenza. The latter require care similar to that required by COVID-19 hospitalizations, but to our knowledge, no insurers waived cost sharing for influenza hospitalizations during the study period. If the presence of out-of-pocket spending for facility services implies the absence of a waiver for these services, a higher proportion of influenza hospitalizations would have out-of-pocket spending for facility services compared with COVID-19 hospitalizations. In this analysis, influenza hospitalizations were those that met similar inclusion and exclusion criteria but had a primary diagnosis of influenza (*ICD-10-CM* diagnosis code J09-J11). None of the influenza hospitalizations included had claims with a COVID-19 diagnosis code (U017).

### Statistical Analysis

We used descriptive statistics to assess patient characteristics, length of hospital stay, and intensive care unit use (eAppendix 2 in the Supplement). To contextualize cost-sharing amounts, we calculated mean and median allowed amounts (reimbursement to providers plus patient liability) across facility and professional and ancillary services among privately insured and Medicare Advantage hospitalizations separately. Analyses were performed using SAS, version 9.4 (SAS Institute Inc).

## Results

### Sample Characteristics

Of 4371 COVID-19 hospitalizations that met the inclusion criteria, 230 were excluded because the insurer was secondary, 63 because data on billing provider type were missing, and 3 because out-of-pocket spending data were missing. Overall, 296 hospitalizations (6.8%) were excluded, leaving 4075 hospitalizations. These hospitalizations occurred among 3875 unique patients; 282 patients had 2 hospitalizations, and 9 patients had 3 hospitalizations. Among the 4075 hospitalizations, 2091 (51.3%) were for male patients; the mean (SD) age of patients was 66.8 (14.8) years. Of these hospitalizations, 1377 (33.8%) were for privately insured patients.

Table 1 gives characteristics of the 4075 hospitalizations. Overall, 1377 hospitalizations (33.8%) were for privately insured patients, and 2698 (66.2%) were for Medicare Advantage patients. Of the former, 825 (59.9%) were for male patients. The mean (SD) length of stay was 7.3 (7.6) days; 640 (46.5%) hospitalizations involved intensive care unit use. Of 2698 hospitalizations for Medicare Advantage patients, 1432 (53.1%) were for female patients. The mean (SD) length of stay was 9.2 (8.9) days; 1212 (44.9%) hospitalizations involved intensive care unit use.

**Table 1.** Characteristics of COVID-19 Hospitalizations Between March and September 2020^a^

Characteristic	Hospitalizations^b^	
	Private insurance	Medicare Advantage
COVID-19 hospitalizations, No.	1377	2698
Month of admission		
March	106 (7.7)	236 (8.7)
April	307 (22.3)	917 (34.0)
May	120 (8.7)	331 (12.3)
June	147 (10.7)	200 (7.4)
July	352 (25.6)	413 (15.3)
August	204 (14.8)	392 (14.5)
September	141 (10.2)	209 (7.7)
Length of stay, mean (SD), d	7.3 (7.6)	9.2 (8.9)
Any intensive care unit use	640 (46.5)	1212 (44.9)
Age, y		
0-17	12 (0.9)	0
18-25	19 (1.4)	1 (0.0)
26-34	75 (5.4)	7 (0.3)
35-44	201 (14.6)	14 (0.5)
45-54	389 (28.2)	101 (3.7)
55-64	550 (39.9)	285 (10.6)
65-74	107 (7.8)	877 (32.5)
75-85	21 (1.5)	925 (34.3)
>85	3 (0.2)	488 (18.1)
Sex		
Female	552 (40.1)	1432 (53.1)
Male	825 (59.9)	1266 (46.9)
Region		
Northeast	200 (14.5)	917 (34.0)
Midwest	315 (22.9)	1062 (39.4)
South	623 (45.2)	433 (16.0)
West	232 (16.8)	274 (10.2)
Plan type		
Health maintenance organization	504 (36.6)	2161 (80.1)
Preferred provider organization	647 (47.0)	512 (19.0)
Consumer-directed health plan	226 (16.4)	0
Unknown	0	25 (0.9)

^a^ Data were obtained from IQVIA PharmMetrics Plus for Academics.

^b^ Data are presented as number (percentage) of patients unless otherwise indicated.

Privately insured hospitalizations were most commonly covered by preferred provider organization plans (47.0%). The mean (SD) allowed amount for privately insured hospitalizations was $42 200 (65 328), and the median was $25 339 (25th-75th percentile, $16 064-$39 484). Medicare Advantage hospitalizations were most commonly covered by health maintenance organization plans (2161 [80.1%]). The mean (SD) allowed amount for Medicare Advantage hospitalizations was $21 501 (21 387), and the median was $17 480 (25th-75th percentile, $14 383-$21 133).

### Out-of-Pocket Spending

Of the 1377 hospitalizations for privately insured patients, 63 (4.6%) had out-of-pocket spending for facility services; and of the 2698 hospitalizations for Medicare Advantage patients, 36 (1.3%) had out-of-pocket spending for facility services. Among these 63 and 36 hospitalizations, mean (SD) total out-of-pocket spending was $3840 ($3186) and $1536 ($1402), respectively; the median total out-of-pocket spending was $3202 (25th-75th percentile, $1836-$5528) and $1223 (25th-75th percentile, $612-$1500). In contrast, out-of-pocket spending for facility services, professional and ancillary services, or both was reported for 981 of the 1377 hospitalizations (71.2%) for privately insured patients and 1324 of the 2698 hospitalizations (49.1%) for Medicare Advantage patients. Among these 981 and 1324 hospitalizations, the mean (SD) total out-of-pocket spending was $788 ($1411) and $277 ($363), respectively; the median total out-of-pocket spending was $329 (25th-75th percentile, $69-$850) and $260 (25th-75th percentile, $109-$300), respectively (Table 2 and Figure). Of all 1377 hospitalizations for privately insured patients, 99 (7.2%) had total out-of-pocket spending greater than $2000 and 34 (2.5%) had total out-of-pocket spending greater than $4000. Of all 2698 hospitalizations for Medicare Advantage patients, 7 (0.3%) had total out-of-pocket spending greater than $2000 and 5 (0.2%) had total out-of-pocket spending greater than $4000.

**Table 2.** Incidence and Magnitude of Out-of-Pocket Spending for COVID-19 and Influenza Hospitalizations^a^

Hospitalization type	Hospitalizations, No. (%)	OOP spending, mean (SD), $		
		Total	Facility services^b^	Professional and ancillary services^b^
**Private insurance, COVID-19 (n = 1377)**				
Had OOP spending for facility services	63 (4.6)	3840 (3186)	3348 (2950)	492 (849)
Had OOP spending for professional and ancillary services	968 (70.3)	768 (1380)	188 (1049)	581 (873)
Had OOP spending for facility services, professional and ancillary services, or both	981 (71.2)	788 (1411)	215 (1107)	573 (869)
**Medicare Advantage, COVID-19 (n = 2698)**				
Had OOP spending for facility services	36 (1.3)	1536 (1402)	1440 (1405)	97 (147)
Had OOP spending for professional and ancillary services	1302 (48.3)	257 (277)	15 (204)	242 (190)
Had OOP spending for facility services, professional and ancillary services, or both	1324 (49.1)	277 (363)	39 (327)	238 (191)
**Private insurance, influenza (n = 61)**				
Had OOP spending for facility services^c^	51 (83.6)	3510 (2524)	2998 (2293)	512 (582)
Had OOP spending for professional and ancillary services^c^	51 (83.6)	3496 (2529)	2907 (2366)	589 (624)
Had OOP spending for facility services, professional and ancillary services, or both	55 (90.2)	3327 (2528)	2780 (2342)	546 (620)
**Medicare Advantage, influenza (n = 178)**				
Had OOP spending for facility services	159 (89.3)	1226 (708)	1117 (665)	109 (144)
Had OOP spending for professional and ancillary services	93 (52.2)	1301 (754)	1072 (747)	229 (145)
Had OOP spending for facility services, professional and ancillary services, or both	173 (97.2)	1150 (728)	1027 (707)	123 (156)

Abbreviation: OOP, out-of-pocket.

^a^ Data were obtained from IQVIA PharMetrics for Academics Database.

^b^ eAppendix 2 in the Supplement gives the codes used to identify facility and professional and ancillary services. Facility services were those billed by hospitals for services such as accommodation. Professional and ancillary services were those billed by clinicians and ancillary providers, such as ambulance providers.

^c^ Although the number of hospitalizations in these 2 rows are the same, they each represent a different set of 51 hospitalizations.

**Figure.**
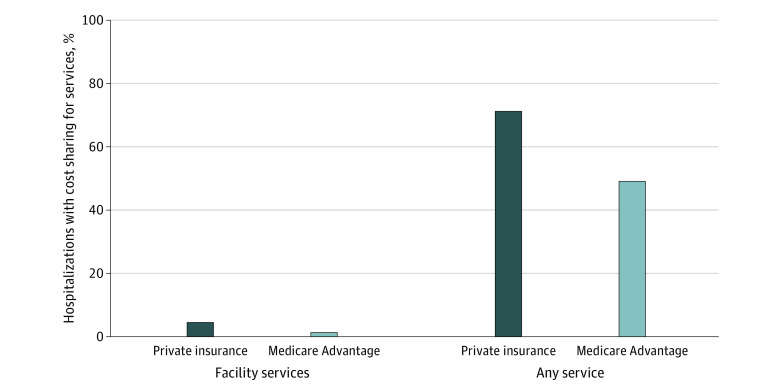
Prevalence of Cost-Sharing Among COVID-19 Hospitalizations for Privately Insured and Medicare Advantage Patients Any service refers to the proportion of hospitalizations with cost sharing for facility services, professional and ancillary services, or both.

Table 3 shows the incidence and magnitude of out-of-pocket spending for each of the 3 main types of professional and ancillary services and for the 4 subtypes of clinician services. Of the 1377 hospitalizations for privately insured patients, 137 (9.9%) had out-of-pocket spending for ambulance services and 918 (66.7%) had out-of-pocket spending for clinician services. When clinician services were analyzed by subtype, 516 (37.5%) hospitalizations had out-of-pocket spending for inpatient evaluation and management services, and 641 hospitalizations (46.6%) had out-of-pocket spending for diagnostic testing services. The mean (SD) out-of-pocket spending for the 516 hospitalizations with out-of-pocket spending for inpatient evaluation and management services was $622 ($765). Compared with hospitalizations for privately insured patients, hospitalizations for Medicare Advantage patients had a higher incidence of out-of-pocket spending for ambulance services (985 patients [36.5%]) but a lower incidence for clinician services (595 patients [22.1%]).

**Table 3.** Incidence and Magnitude of Out-of-Pocket Spending for Professional and Ancillary Services Among COVID-19 Hospitalizations^a^

Service type	Privately insured (n = 1377)				Medicare Advantage (n = 2698)			
	Patients with ≥1 claim, No. (%)	OOP spending per patient overall, mean (SD), $	Patients with OOP spending, No. (%)	OOP spending among patients with OOP spending, mean (SD), $	Patients with ≥1 claim, No. (%)	OOP spending per patient overall, mean (SD), $	Patients with OOP spending, No. (%)	OOP spending among patients with OOP spending, mean (SD), $
Main types of professional and ancillary services^b^								
Ambulance	305 (22.1)	59 (248)	137 (9.9)	596 (550)	1425 (52.8)	87 (139)	985 (36.5)	239 (130)
Clinician	1334 (96.9)	317 (682)	918 (66.7)	476 (789)	2608 (96.7)	29 (117)	595 (22.1)	130 (221)
Miscellaneous^c^	272 (19.8)	32 (177)	167 (12.1)	263 (445)	401 (14.9)	1 (10)	99 (3.7)	26 (44)
Subtypes of clinician services								
Emergency department	746 (54.2)	31 (103)	399 (29.0)	106 (169)	1493 (55.3)	0 (1)	255 (9.5)	2 (2)
Inpatient evaluation and management	1234 (89.6)	233 (557)	516 (37.5)	622 (765)	2495 (92.5)	24 (103)	394 (14.6)	162 (225)
Inpatient diagnostic	668 (48.5)	36 (85)	641 (46.6)	78 (111)	1438 (53.3)	3 (14)	427 (15.8)	18 (32)
Other inpatient^d^	109 (7.9)	17 (179)	63 (4.6)	375 (757)	314 (11.6)	2 (19)	83 (3.1)	67 (87)

Abbreviation: OOP, out-of-pocket.

^a^ Data were obtained from IQVIA PharMetrics for Academics Database.

^b^ Professional and ancillary services include those submitted by clinicians and those from ancillary service providers, such as ambulance providers. eAppendix 2 in the Supplement gives details.

^c^ Services from miscellaneous providers, such as durable medical equipment providers.

^d^ Includes services submitted by clinicians with a hospital place of service but no procedure code for evaluation and management or diagnostic services (eg, procedures, anesthesia).

### Analyses Assessing Presence of Cost-Sharing Waivers

Among hospitalizations for privately insured and Medicare Advantage patients in the lowest quartile of out-of-pocket spending before hospitalization, the proportion with out-of-pocket spending for facility services was 8.3% for privately insured patients and 1.8% for Medicare Advantage patients (eAppendix 4 in the Supplement). Sixty-one influenza hospitalizations for privately insured patients and 178 influenza hospitalizations for Medicare Advantage patients met the inclusion criteria. Of these hospitalizations, 51 (83.6%) for privately insured patients and 159 (89.3%) for Medicare Advantage patients had out-of-pocket spending for facility services compared with 63 (4.6%) among COVID-19 hospitalizations covered by private insurance and 36 (1.3%) covered by Medicare Advantage plans (Table 2). In the sensitivity analysis including claims for unclassified services that did not meet the criteria for a facility or professional or ancillary service, the results were not substantially different from those of the main analysis.

## Discussion

In this cross-sectional study of COVID-19 hospitalizations in the US between March and September 2020, the incidence of out-of-pocket spending differed substantially for facility and professional and ancillary services. Few COVID-19 hospitalizations had out-of-pocket spending for facility services billed by hospitals. However, 71.2% of hospitalizations for privately insured patients and 49.1% of hospitalizations for Medicare Advantage patients had out-of-pocket spending for facility services, services billed by clinicians and ancillary service providers, or both. If the absence of out-of-pocket spending for facility services is an indicator of the presence of an insurer cost-sharing waiver for these services (an assumption supported by our analyses), most study hospitalizations were covered by insurers that at least waived cost sharing for facility services. If this was true, the high incidence of out-of-pocket spending for professional and ancillary services suggests that many insurer cost-sharing waivers may have failed to capture all hospitalization-related care.

Whether this failure was intentional is unclear. In contrast to COVID-19 testing and vaccination, there is no federal mandate for insurers to waive cost sharing for COVID-19 hospitalizations.^13^ Consequently, insurer waivers could be heterogeneous, with some applying only to facility services and others applying to hospitalization care more broadly. Even if insurers intend for waivers to capture all hospitalization-related care, implementation problems may occur. For example, patients may be billed erroneously if insurers do not link clinician inpatient evaluation and management bills to the COVID-19 hospitalization.

Insurers and clinicians might consider 3 steps to mitigate patient financial liability for professional and ancillary services related to COVID-19 hospitalizations. First, insurers with waivers of limited scope could consider implementing a comprehensive waiver, such as one that covers all services on or between the admission and discharge dates of hospitalizations. Second, insurers that already have comprehensive waivers could work to ensure appropriate implementation. Third, clinicians could encourage patients to contest any bills for professional and ancillary services that should be covered under an insurer’s cost-sharing waiver.

In this study, 4.6% of hospitalizations for privately insured patients and 1.3% of hospitalizations for Medicare Advantage patients had out-of-pocket spending for facility services. Among these hospitalizations, the mean total out-of-pocket spending was $3840 for privately insured patients and $1536 for Medicare Advantage patients. If the presence of out-of-pocket spending for facility services implies the absence of an insurer cost-sharing waiver for these services, as suggested by the finding that most influenza hospitalizations had cost sharing for facility services, our findings suggest that the out-of-pocket burden for COVID-19 hospitalizations could be large without insurer cost-sharing waivers. This would have important policy implications. As of August 2021, 72% of the 2 largest private insurers in each state no longer waive cost sharing for COVID-19 hospitalizations. Furthermore, several large Medicare Advantage insurers have allowed cost-sharing waivers to expire.^14^ Analyses suggest that patients covered by these insurers may now experience substantial financial burden for COVID-19 hospitalizations, particularly those who are privately insured.

Although not the primary focus of this study, we report some of the first estimates of health care spending for COVID-19 hospitalizations for privately insured and Medicare Advantage patients. Our estimates indicate that mean spending for privately insured patients ($42 200 per hospitalization) was twice as high as spending for Medicare Advantage patients ($21 387 per hospitalization). This latter total is comparable to spending for fee-for-service Medicare beneficiaries hospitalized for COVID-19.^15^

### Strengths and Limitations

This study has strengths. We used a national database that includes both privately insured and Medicare Advantage plans. These plans are important sources of coverage for adults aged 50 and older, a group that is at high risk for COVID-19 hospitalization.^16,17,18^

This study also has limitations. First, we cannot prove that COVID-19 hospitalizations in this study were mostly covered by plans with cost-sharing waivers. Second, if patients did not pay the amounts they were billed or were not billed because they died in the hospital, the incidence of actual out-of-pocket spending would differ from the incidence estimated by this study. However, the amount billed to patients still shows the financial burden patients may experience without cost-sharing waivers. Third, the number of hospitalizations with out-of-pocket spending for facility services was small, likely owing to the widespread presence of insurer cost-sharing waivers during 2020. Consequently, mean total out-of-pocket spending among these hospitalizations may be imprecisely estimated. Fourth, our sample of 4075 COVID-19 hospitalizations represents a small proportion of the roughly 311 000 hospitalizations in the US from March to September 2020.^19^ Thus, results may not necessarily be generalizable to all privately insured and Medicare Advantage patients. However, most hospitalizations of privately insured patients in our study were covered by preferred provider organization plans, and most hospitalizations of Medicare Advantage patients were covered by health maintenance organizations, consistent with the national distribution of plan types among privately insured and Medicare Advantage enrollees.^16,20^ Fifth, findings on out-of-pocket spending may not be generalizable to traditional Medicare enrollees. For lengthy hospitalizations, such as those for patients with COVID-19 infection, cost sharing is typically lower for traditional Medicare enrollees compared with Medicare Advantage enrollees.^21^

## Conclusions

The findings of this cross-sectional study suggest that insurer cost-sharing waivers for COVID-19 hospitalizations may not always capture all hospitalization-related care. Moreover, patient financial burden for COVID-19 hospitalizations could be substantial without insurer waivers. The increasing trend toward abandonment of these waivers suggests that relying on voluntary actions by insurers is not an ideal strategy if policy makers wish to protect patients from the costs of COVID-19 hospitalizations.^9,10^ To achieve this goal, federal policy makers might consider legislation mandating insurers to waive cost sharing for COVID-19 hospitalizations throughout the public health emergency.^11^ Such a mandate would ideally include all hospitalization-related care, similar to existing federal mandates that require insurers to fully cover all direct and related costs of COVID-19 testing and vaccines.^13^ Future research should include monitoring of patient financial burden resulting from COVID-19 hospitalizations as coverage policies change.

## Notes

Supplement. **eAppendix 1.** Rationale for Including Hospitalizations With Admission and Discharge Date Before Instead of Through September 30, 2020 **eAppendix 2.** Codes Used in Analyses **eAppendix 3.** Sensitivity Analysis Including Unclassified Claims **eAppendix 4.** Incidence of Cost-Sharing for Facility Services Among Hospitalizations for Patients in the Lowest Quartile of Out-of-Pocket Spending Prior to Hospitalization
